# The genomic diversity and spatial patterns of *Mycobacterium bovis* in Ireland revealed by whole genome sequencing

**DOI:** 10.1186/s13620-025-00324-0

**Published:** 2025-12-03

**Authors:** James O’Shaughnessy, Nicola Harvey, Brian Byrne, Máire McElroy, Montserrat Gutierrez, Declan Murphy, Kevin Kenny, Henrietta Cameron, Deirdre Prendergast, Rebecca Cupial, Margaret Goggin, Lionel Kenneth Dygico, Jordy Smith, Jamie A. Tratalos, Ryan Devaney, Purnika Ranasinghe, Tara Ardis, Adrian Allen, Guy McGrath, Stephen V. Gordon, Damien Farrell

**Affiliations:** 1 Department of Agriculture, Food and the Marine Laboratories, Backweston, Celbridge, Co. Kildare Ireland; 2 Centre for Veterinary Epidemiology and Risk Analysis, School of Veterinary Medicine, University College Dublin, Belfield, Dublin 4, Ireland; 3 School of Veterinary Medicine, University College Dublin, Belfield, Dublin 4, Ireland; 4 Agri-Food and Biosciences Institute (AFBI), Large Park, Hillsborough Co. Down , BT26 6DR UK; 5 One Health Centre, University College Dublin, Belfield, Dublin 4, Ireland; 6 UCD Centre for Experimental Pathogen Host Research, University College Dublin, Dublin 4, Ireland

**Keywords:** Mycobacterium bovis, Bovine tuberculosis, Whole genome sequencing, Ireland

## Abstract

**Background:**

Bovine tuberculosis (bTB), caused primarily by *Mycobacterium bovis*, remains a major challenge across the island of Ireland. Despite decades of an eradication programme that encompasses cattle testing, movement restrictions, and badger culling, bTB prevalence has increased in recent years. The epidemiology of bTB is complex, with inter-species (e.g. badger-cattle) and intra-species (e.g. cattle-cattle) transmission of infection. This study utilised whole-genome sequencing (WGS) to investigate the genetic diversity and spatial distribution of *M. bovis* across the island of Ireland as a route to help elucidate transmission of infection.

**Results:**

A total of 5,875 *M. bovis* isolates from cattle and badgers were analysed to identify strain diversity, geographic clustering, and patterns of strain sharing within and between host species. Our findings reveal significant regional variation in strain distribution, with certain clades predominantly confined to specific regions, while others are more widely dispersed. Strong genetic similarities between cattle and badger isolates support the role of badgers as infection reservoirs. Furthermore, a subset of herds contained multi-strain infections and amongst these herds there were ‘controlled finishing units’ (CFUs), where infection was more likely driven by inward cattle movements than local transmission.

**Conclusions:**

By integrating phylogenetic analysis with spatial mapping and cattle movement data, this study provides new insights into *M. bovis* transmission pathways and highlights the value of WGS in refining Ireland’s bTB control strategies.

**Supplementary Information:**

The online version contains supplementary material available at 10.1186/s13620-025-00324-0.

## Introduction

Bovine tuberculosis (bTB), caused by members of the *Mycobacterium tuberculosis* complex particularly *Mycobacterium bovis*, continues to threaten livestock health and agricultural productivity in Ireland and globally. Despite an extensive eradication programme dating back to the 1950 s, the island of Ireland still incurs a substantial annual cost in managing bTB outbreaks. During 2023, the total programme costs in the Republic of Ireland were €108.4 million, with €74 million paid by the Exchequer [1]. Costs exceeded £55 million in Northern Ireland in 2023/24. Spread of bTB is shaped by its complex epidemiology, with a risk of transmission between cattle and wildlife reservoirs such as badgers [2]. Cattle movement patterns play an important role in the dissemination of *M. bovis* in Ireland. Ireland’s livestock industry involves extensive trade [3], with millions of animals transported annually between farms and through livestock marts. Studies, such as Tratalos et al. [4], have demonstrated the spatial and network characteristics of these movements, revealing how they contribute to the spread of infectious diseases. The role of wildlife, particularly badgers, in sustaining *M. bovis* transmission is well documented, leading to long-standing efforts to reduce spillover risk through targeted culling and, more recently, badger vaccination programs [5]. The epidemiological role of deer remains under explored, despite their increasing density and range in Ireland [6, 7]. In RoI, deer populations in County Wicklow have been suggested to be a source of infection, and there is evidence from Northern Ireland suggesting deer may not be simple, dead-end spillover hosts [8], although the latter may be context dependent. There is a need for a more refined, risk-based approach to bTB control [9] that integrates genomic surveillance to enhance movement tracking and targeted interventions.

Advances in genomic tools, including whole-genome sequencing (WGS), offer unprecedented resolution to aid in understanding the transmission dynamics of *M. bovis*. Identification of single nucleotide polymorphisms (SNPs) across the genome by alignment against a reference genome is the highest resolution method of delineating strains [10]. Compared to traditional *M. bovis* molecular typing methods such as spoligotyping and variable-number tandem repeat (VNTR) analysis, WGS provides greater discriminatory power [11], allowing researchers to resolve transmission networks and identify fine-scale associations of specific molecular types with geographic regions. WGS has been successfully applied in several bTB-endemic regions, helping to differentiate local persistence from new introductions and track cross-border transmission. In Great Britain, for example, WGS has been used to investigate the role of badgers in *M. bovis* transmission, demonstrating that direct transmission between cattle and badgers occurs frequently [2]. Similarly, in Northern Ireland, genomic epidemiology has helped clarify the relationship of *M. bovis* between sympatric cattle and badger populations, highlighting the complexity of multi-host disease maintenance [12]. Beyond wildlife transmission, WGS has also been instrumental in assessing the impact of cattle trade on disease dissemination, with studies in Spain [13, 14] using genomic data to track the movement of *M. bovis* across regional borders. By leveraging these genomic approaches in Ireland, we can enhance bTB surveillance, refine risk-based control strategies, and improve outbreak investigations to ultimately accelerate eradication efforts.

A challenge in eradication efforts is the presence of residual infection - undetected *M. bovis* carriers that persist within herds despite testing and movement restrictions [15]. The main diagnostic tool in Ireland, the single intradermal comparative tuberculin test (SICTT), has limitations in sensitivity [16], leading to the potential for infected animals to remain in herds or be moved undetected to new locations [1]. There is also the potential for undetected transmission due to the time gap between diagnostic tests [17]. This contributes to prolonged outbreaks and re-emerging infections, complicating disease control. WGS will be a valuable tool to address such persistent infection by offering high-resolution strain identification, enabling researchers to distinguish between reactivation of local infections and new introductions of *M. bovis* into a herd. Hence by integrating WGS data with movement records and epidemiological investigations it should be possible to determine whether breakdowns are due to residual infection within a herd, transmission from neighbouring farms, or long-distance spread through cattle movements.

Controlled Finishing Units (CFUs) are specific beef finishing herds designated by the Irish government Department of Agriculture Food and the Marine (DAFM). These are non-breeding herds where all cattle are destined for direct slaughter. The conditions permit bTB-restricted CFUs to purchase cattle for direct slaughter, including test-negative animals from restricted herds. CFUs are intended to have strict bio-security measures to prevent potential spillover to neighbouring herds or wildlife. To minimize the potential for spread to contiguous holdings, the CFU production system must be one of the following: (1) The cattle must be permanently housed (zero grazed), or (2) there are no contiguous cattle herds, or (3) The pasture is walled, double fenced or equivalent to prevent nose-to-nose contact with cattle on a contiguous holding (all grazing fragments must be fenced). While CFUs aim to mitigate the spread of bTB by isolating potentially infected animals, their growing prominence raises concerns about disease management. CFUs have become increasingly integral to Ireland’s beef production, accounting for 458,500 out of 1,227,620 cattle slaughtered in 2024 [18]. As CFUs handle an increasing proportion of cattle, their potential role in the epidemiology of bTB becomes more important. In this work we examined the *M. bovis* strains found in these CFU herds and the implications for bTB control from a risk perspective.

A first step towards a genomics-based approach to inform bTB control is to establish the genetic diversity of *M. bovis* circulating in Ireland. Therefore, we present herein the findings of a large-scale genomic study of *M. bovis* across the island of Ireland, integrating WGS data with spatial mapping. By analysing 5,875 *M. bovis* isolates collected from cattle and badgers, this study provides a high-resolution view of strain diversity and geographic clustering. We characterize regional strain distributions, typical patterns of local spread, and assess the extent of strain-sharing between cattle and badgers. This work confirms the existence of a single major evolutionary lineage of *M. bovis* (Eu1 clonal complex) across Great Britain and the island of Ireland as described by Allen et al. [19], indicating historical homogenization of the pathogen population. We also take a preliminary look at herds with multi-strain infections such as in CFUs. These insights gleaned from this study highlight the potential contribution of WGS for optimizing Ireland’s bTB eradication strategies with future risk-based control approaches.

## Methods

### Sampling and isolate collection

#### Republic of Ireland (RoI)

An archive of tissues confirmed to be tuberculous by histopathologic examination was established at DAFM laboratories in 2013. Culture and isolate recovery from these tissues, along with recovery of frozen isolates, provided material for early sequencing runs. Subsequent to this, *M. bovis* isolates from ‘homebred’ cattle from across the RoI (dating from 2017 to mid-2022) were systematically prioritised for sequencing. The aim was to produce a baseline national map of sequenced isolates. Using data provided by the DAFM Animal Health Computer System (AHCS), homebred cattle were categorised as animals that never left the farm of origin during the period from birth until two weeks prior to slaughter. Given that some cattle, both homebred and not, are sold in livestock markets in the period shortly before slaughter, our approach was to use the herd location coordinates of the herd the cattle resided in two weeks prior to slaughter, and not the herd number of the animal at slaughter, to better reflect their true location. Isolates from individual farms or areas of particular interest, such as part of local epidemiological investigations, were also included in our analysis. These isolates extended the temporal range of our dataset up to mid-2024. Additional isolates were obtained from badgers as part of a parallel badger surveillance program. All RoI samples had been cultured and archived at the DAFM Laboratories.

#### Northern Ireland (NI)

An initial focus for sampling efforts was directed towards small/discrete regions within the six counties of NI in which specific research questions needed to be answered. The Co. Down site of the Test Vaccinate or Remove (TVR) project from 2014 to 2018 [12] was one such area, as was the border region with Co. Monaghan, for the purposes of assessing cross border disease dissemination. A general shift to more NI wide sampling across wider geographic and temporal ranges is now underway to define historic and extant diversity alongside clade/strain core ranges [20]. To assist with this effort, the Agri-Food and Biosciences Institute (AFBI) uses tissues confirmed as affected by tuberculous pathology, from contemporary herd breakdowns, road traffic accident badger carcases and other wildlife sources, identified by the NI bTB eradication scheme in NI. For the cattle population, AFBI will in future aim to sequence at least one disclosing *M. bovis* isolate per breakdown herd. Alongside this effort, AFBI has a frozen archive of TB confirmed isolates from every herd breakdown occurring over the previous 20 years, with a subset of these from 2009 to 2019 having been collected at the animal level. All isolates are cultured/re-cultured using methods previously described [12].

#### Great Britain (GB)

In addition to samples from the island of Ireland, we included 91 representative genome sequences isolated in GB, generated by the Animal and Plant Health Agency (APHA). These data are available on the SRA under bioproject PRJEB40340.

### DNA extraction, library preparation and sequencing

#### RoI

Following routine culture of tissue samples for *M. bovis*, a single colony of *M. bovis* was selected and subsequently sub-cultured on modified Middlebrook 7H11 medium (7H11 EO labs, supplied by Syntec Ireland) at 37 °C. Once a bacterial lawn was established (up to 7 weeks), the propagated cells were washed with 1 ml of UltraPure™ distilled water and transferred by pipette into a 2 ml Eppendorf tube. The isolates were heat killed for 1 h at 80 °C, centrifuged at 10,000 RPM for 10 min, and the supernatant was discarded. The resulting pellet was reconstituted in 100 µl Lysozyme (10 mg/ml, Sigma-Aldrich, Germany) and incubated for 60 min at 37 °C with vortexing after 30 min. A 100 µl volume of bacterial lysis buffer (MagNA Pure 96 bacterial lysis buffer, Roche diagnostics, Dublin, Ireland) and 20 µl Proteinase K (19 mg/ml, Roche Diagnostics) was added, the suspension vortexed and incubated for 60 min at 65 °C followed by incubation for 10 min at 95 °C. DNA extraction was performed with 200 µl of the suspension using the MagNA Pure 96 system (Roche Diagnostics as previously described [21, 22]).

DNA Libraries for each *M. bovis* isolate were prepared using the Illumina DNA Prep kit (Illumina, Netherlands) as per the manufacturer’s instructions using 30 µl of extracted DNA and the Hamilton NGS STAR robotic liquid handler (Hamilton, Birmingham, UK). The tagmented DNA was amplified in a working volume of 50 µl with the following settings: heated lid at 100 °C, initial cycle at 68 °C for 3 min followed by 98 °C for 3 min and 5 cycles of (98 °C for 45 s, 62 °C for 30 s and 68 °C for 2 min) with a final run at 68 °C for 1 min followed by a hold temperature of 10 °C (Hamilton NGS STAR on-deck thermal cycler). A pooled amplified library (PAL) was prepared by adding 5 µl of each amplified library to a sterile DNA and RNA free 1.5 ml tube. The PAL was quantified using a Qubit dsDNA HS Assay Kit (Thermo Scientific) and diluted in resuspension buffer with Tween20 (RSB; Illumina, Netherlands) to give a final loading concentration of 750 pM. The PAL was spiked with 2% PhiX control (Illumina, Netherlands) and sequenced on a NextSeq2000 (Illumina, Netherlands) using a P1 300-cycle (2 × 150 bp) cartridge and P1 flow cell (Illumina, Netherlands.). The Q30 (≥ 85%) and error rate (≤ 6%) of each sequencing run was assessed in BaseSpace (Illumina, Netherlands).

A small subset of RoI samples cultured at DAFM Laboratories were subsequently sequenced at UCD Conway Institute Genomics Core. DNA extraction and library preparation at UCD was performed as detailed in Crispell et al. [6].

#### NI

AFBI isolates were DNA extracted using the cetyl hexadecyl trimethyl ammonium bromide (CTAB) and solvent extraction protocols at the AFBI Pathogen Genomics Laboratory of the Bacteriology Branch, AFBI Stormont, Belfast. Sequencing libraries with 500–600 bp inserts were generated using the Illumina DNA Prep workflows and sequenced on an Illumina Miseq instrument using V2 chemistry, producing paired end reads of ~ 250 bp.

### Variant calling and phylogenetic analysis

WGS reads were analysed using SNiPgenie version 0.7.0, a microbial variant-calling and phylogenetic analysis tool [23] (available at https://github.com/dmnfarrell/snipgenie). Briefly, reads were aligned to the *M. bovis* AF2122/97 reference genome [24] using the Burrows-Wheeler aligner (BWA) [25]. SNP variants were identified by bcftools [26]. Variants were accepted if they had a quality score of at least 40 (QUAL >= 40), a minimum read depth of 30 and at least four reads had to support the variant across both strands (DP4 ≥ 4). Variant positions were further filtered to mask annotated repeat regions (those encoding proline-glutamic acid (PE) and proline-proline-glutamic acid (PPE) proteins [27]). The variant base pairs for all samples were concatenated to construct a sequence alignment for phylogenetic analysis. A maximum-likelihood (ML) phylogenetic tree was generated using RAxML [28] with the generalized time-reversible (GTR) substitution model [29]. and 100 bootstrap replicates. The resulting support values were used to assess branch confidence on major clades. As part of QC samples were checked for coverage and percentage of reads mapped. Those with anomalous values were also run through Kraken (version 2.12) [30] to check for contaminants.

Spoligotypes were inferred from sequencing reads using custom Python code available in SNiPgenie [23]. Briefly, up to 3 million reads were subsampled and converted to FASTA format. A BLAST database [31] was created from the reads, and known direct repeat (DR) spacers were aligned to this database using BLAST with an e-value threshold of 0.1. Spacers were considered present if they had at least two hits with >90% query coverage and ≤ 2 mismatches. A 43-digit binary spoligotype pattern was generated based on the presence (1) or absence (0) of each standard spacer. The resulting binary spoligotype was then matched to the Mbovis.org reference database [32] to assign the corresponding SB number where available.

### Global phylogeny

Globally representative *M. bovis* genome sequences using metadata provided by Zwyer et al. [10] were downloaded from the Sequence Read Archive (SRA) and combined with a representative set of 72 genomes from our Irish dataset (representing every strain within each major clade). These were aligned against the *M. tuberculosis* H37Rv sequence and a phylogeny was built as described above. Zwyer lineage assignments for the Irish genomes were identified using TBProfiler [33].

### Metadata and location data

Metadata on herd locations, cattle movements, and badger sett locations were obtained from DAFM and curated by the UCD Centre for Veterinary Epidemiology and Risk Analysis (CVERA). Herd locations were derived from land-parcel data linked to each sampled herd, as available through the DAFM Land Parcel Identification System (LPIS). Locations for herds were calculated as the centroid of the largest parcel in each herd. Badger locations were provided as coordinates of the nearest sett to where the animal was trapped.

### Movement data

RoI cattle movement records were extracted from the Animal Identification and Movement (AIM) system, covering all sampled animals, as well as those connected through movement networks during the study period. This included all movements prior to the animals’ final transport to slaughter. Movement data records the origin and destination of the movement. The origin is a herd in all situations except for imports or movements from a market (known as ‘marts’ in RoI). The destination can be a herd, a market, a country in the case of exports, or a slaughterhouse. Spatial locations of movements were extracted from the coordinates of each herd, also obtained from the LPIS database. For the purposes of this study mart locations that the animal passed through were ignored. Animal movements from NI were not available. Movements were extracted in Python by querying the large table out of memory using Polars [34] lazy evaluation and then converted to a Pandas DataFrame for further processing.

### Visualization and mapping

Phylogenetic trees were visualized using the R packages ggtree [35] and gheatmap to annotate metadata, including herd location and clade assignment. All geospatial analyses of herd and sett locations were performed using the Python package GeoPandas [36] and associated libraries. Spatial data was converted to the Irish Grid Reference System (TM 75). Custom visualisations were created in Jupyter notebooks [37] to integrate phylogenetic and spatial data, enabling detailed exploration of geographic clustering and movement trends.

### Strain nomenclature

A three level hierarchical population structure was determined from the phylogeny using the fastbaps [38] package in R. The top level is classified as clades (or major clades) in the text. The lowest level is referred to as the strain. Only the major clades (numbers from 1 to 18) are labelled explicitly in the text. For analysis purposes ‘clusters’ were defined within each strain by applying a SNP distance threshold (≤ 12 SNPs) using the SNP distance matrix.

### Selection of representative isolates

For selecting a subset of 600 tips for the representative phylogeny we implemented a custom R function to reduce phylogenetic redundancy by subsampling the input tree to a user-defined number of representative taxa. The function computes pairwise cophenetic distances, applies hierarchical clustering to group taxa into k clusters, and retains a single representative tip from each cluster. The resulting pruned tree maintains broad phylogenetic diversity while limiting overrepresentation of closely related taxa. This code is available in the SNiPgenie github repository at https://github.com/dmnfarrell/snipgenie.

### Spatial binning of clades

Spatial binning of samples in the same clade was calculated in GeoPandas by binning points over a hexagonal grid and retaining all those polygons that had at least one point from each clade. Outliers were first removed by excluding those points greater than 3 standard deviations from the centroid of the points in each clade. The number of points in each bin were recorded. The same method is used for the home ranges as described below.

### Home range identification

“Home ranges”, i.e. geographical localisation of particular *M. bovis* strains, were identified by using the location of isolates from (a) badger setts and (b) sampled herds with no inward movements of cattle in the last 5 years, or no movements of cattle from herds with positive tests in the same time period. The rationale was that these isolates most likely result from local transmission events and thus be considered locally spreading in the area. Isolate locations were grouped by strain, with any group containing < 5 members not considered. For each group of points outliers were removed unless they were badgers (since badger strains will always represent locally circulating strains). Finally for each group we mapped the points onto a predefined grid of hexagonal bins, counting the number of points in each bin. This grid then defines the extent of each strain. Multiple strains often overlap in the same grid coordinates and are stored as separate entries.

### Calculation of CFU association with local strains

A list of herds that now have (or had during the time of sampling) CFU status were extracted from the AHCS database. The sampled herds were checked against this list and the entire set of samples were assigned CFU status (true/false). To detect degree of neighbourhood strain sharing for all herds we took the centroid of each herd (from the largest parcel) and detected any points (herd or sett location) within a threshold distance. Various metrics were recorded for each herd during this procedure: number of neighbours with strains, total number of inward moves since 2018, moves of animals in herd with an isolate, median distance of these moves and whether a farm is a CFU. These were tabulated into a table for statistical analysis and regression. Logistic regression was performed using the Python statsmodels package [39]. We could also visualise the herd in question and its neighbouring points coloured by strain(s).

## Results

### Dataset

**Fig. 1:**
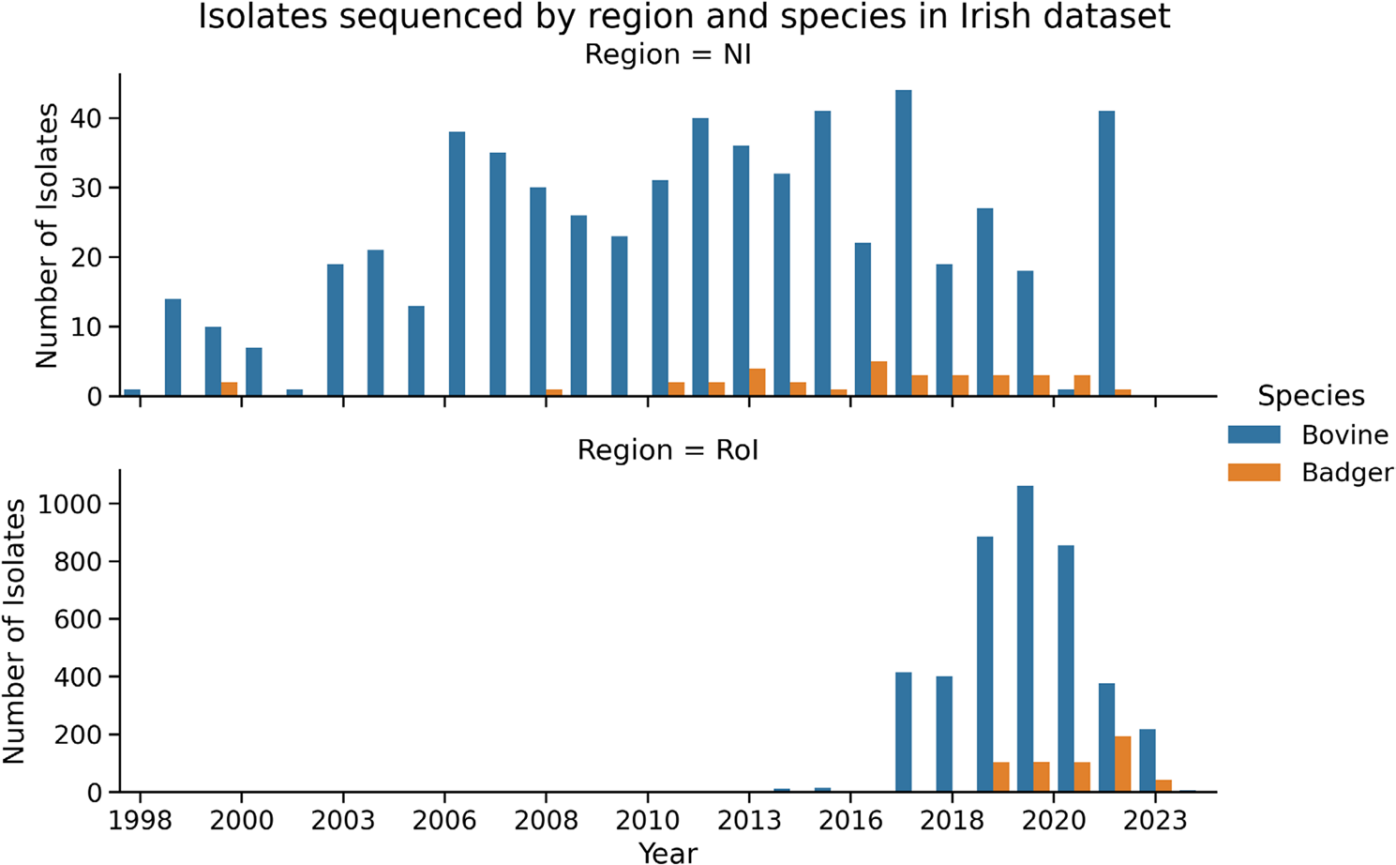
Bar plots showing the number of Irish M. bovis samples sequenced for this study by year (x-axis) and coloured by species. RoI (lower plot) denotes samples from the Republic of Ireland, while NI (upper plot) are samples collected in Northern Ireland. T*h*e relatively few samples from other species are not included here for clarity

**Fig. 2:**
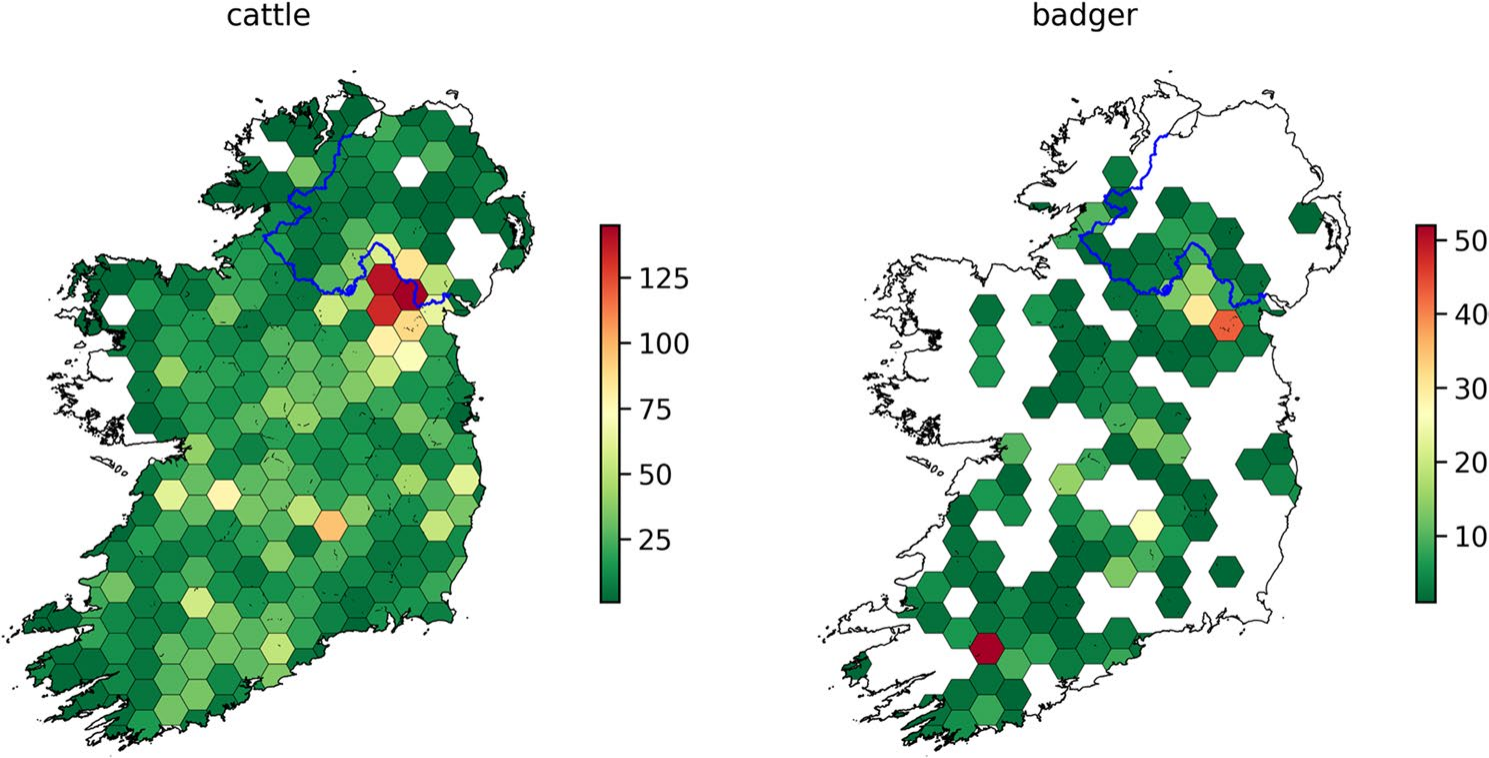
Hex bin plot showing the coverage of sequenced Irish isolates across the country for bovine (left) and badger (right). Each bin represents the number of isolates in that area as indicated in the color bars on right. The RoI/NI border is indicated by the blue line

*M. bovis* isolates were obtained from cattle herds covering the entire island of Ireland (RoI and NI), including all 32 counties. Currently the RoI dataset consists of 5,135 sequenced isolates (4342 bovine, 567 badger and the remainder a mix of other species). There were 2,962 unique RoI herds sampled, with 649 additional sequences covering NI (597 bovine, 35 badger and the remainder deer and ovine). The distribution of samples by time, species and region is shown in Fig. 1. Sampling was not uniform across the island of Ireland, rather there was a particular focus on counties with high bTB prevalence e.g. counties Monaghan, Cork and Kilkenny. Spatial density of sampling is shown in Fig. 2. 65% of RoI cattle isolates were from animals considered to be homebred.

### Whole genome sequencing

High-quality sequencing data was generated for 5,135 *M*. *bovis* isolates from the RoI. On average, each genome had 99.84% coverage (lower 2.5 %, 0.9939; upper 97.5 %, 0.9999) of the reference genome with a minimum coverage of 98.57%. The mean read depth was 113 reads with a minimum read depth ≥ 20 reads. Mean read depth was 266 at the 97.5% percentile with few samples above this. It was not considered necessary to normalise reads [40].

### Phylogeny and strain diversity

**Fig. 3:**
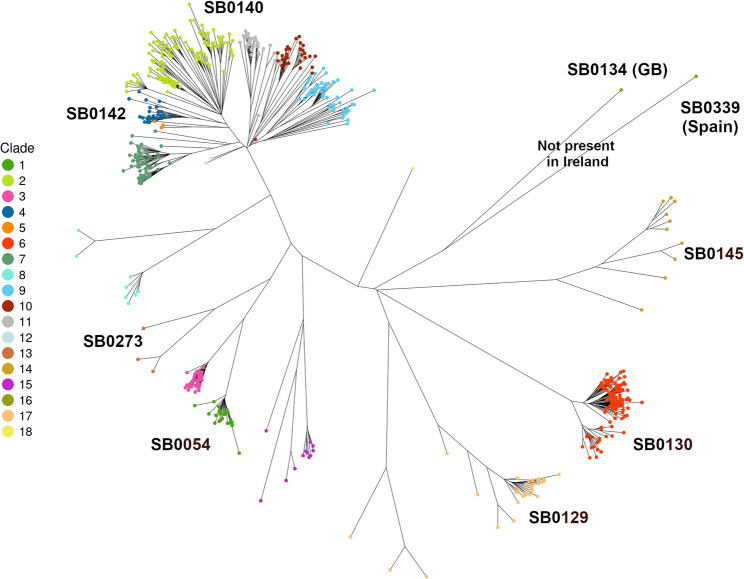
Unrooted phylogeny indicating the major clades of M. bovis found in Ireland. Tips are coloured by their clade and some clades are labelled by the associated spoligotype. This is a representative phylogeny of 600 samples that covers the entire diversity seen

The phylogeny shown in Fig. 3 uses 600 representative isolates and reflects the full diversity from our island-wide sampling (a complete tree is given in Figure S1). Globally, all Irish strains fall within the Eu1 (La1.8.1) clonal complex [19] as defined by Zwyer et al. [10] (see Figure S3).

The maximum SNP distance between any two isolates is 491 SNPs (median SNP distance = 242). Eighteen major clades were identified but three [12, 16, 18] have less than 5 members (isolates). The structure of the tree indicates two distinct clade groups. One group (top of Fig. 3) of more closely related clades constitutes the majority of all isolates (66%) and the remainder are a group of more distantly related clades that are generally less well represented. Of these clades 5 and 16 appear to be GB strains that have few or no representatives in Ireland. There are no GB samples in clade 6; the GB and NI samples are otherwise represented in most clades. Figure S2 illustrates the placement of all GB samples in the reduced phylogeny. Only one clade, 13 (*n* = 46), appears largely confined to NI (though there is a cluster in County Donegal). Most of the remaining samples in NI belong to clades 2 and 9. The average genetic distance between clades is 283 SNPs excluding the 3 clades with < 5 members and 254 SNPs when we also remove three of the most distantly related outlier clades [6, 14, 17]. The minimum SNP distance is 100.

Most common spoligotypes are labelled in Fig. 3. Spoligotypes are predominantly SB0140 (50%), SB0142 (11%) and SB0130 (11%). SB0054 is notable as being uniquely confined to Co. Wicklow (clade 1 in the phylogeny). Clade 16 is not present in Ireland and represents a single Sample from Spain with spoligotype SB0339 (provided by the European Reference Laboratory for Bovine Tuberculosis (EURL TB)). This clade also includes three GB samples with spoligotype SB0134. This clade has been included since it provides a convenient out-group for the all-Ireland phylogeny.

Strains were defined at the lowest level of the fastbaps hierarchy. The number of strains in each clade ranges widely from 1 to 55 depending on clade size and diversity. Typically, strains represent a maximum distance of 20–30 SNPs between members, but often much less. Some strains are more common, with the largest having 280 samples.

### Geographic spread of clades

**Fig. 4:**
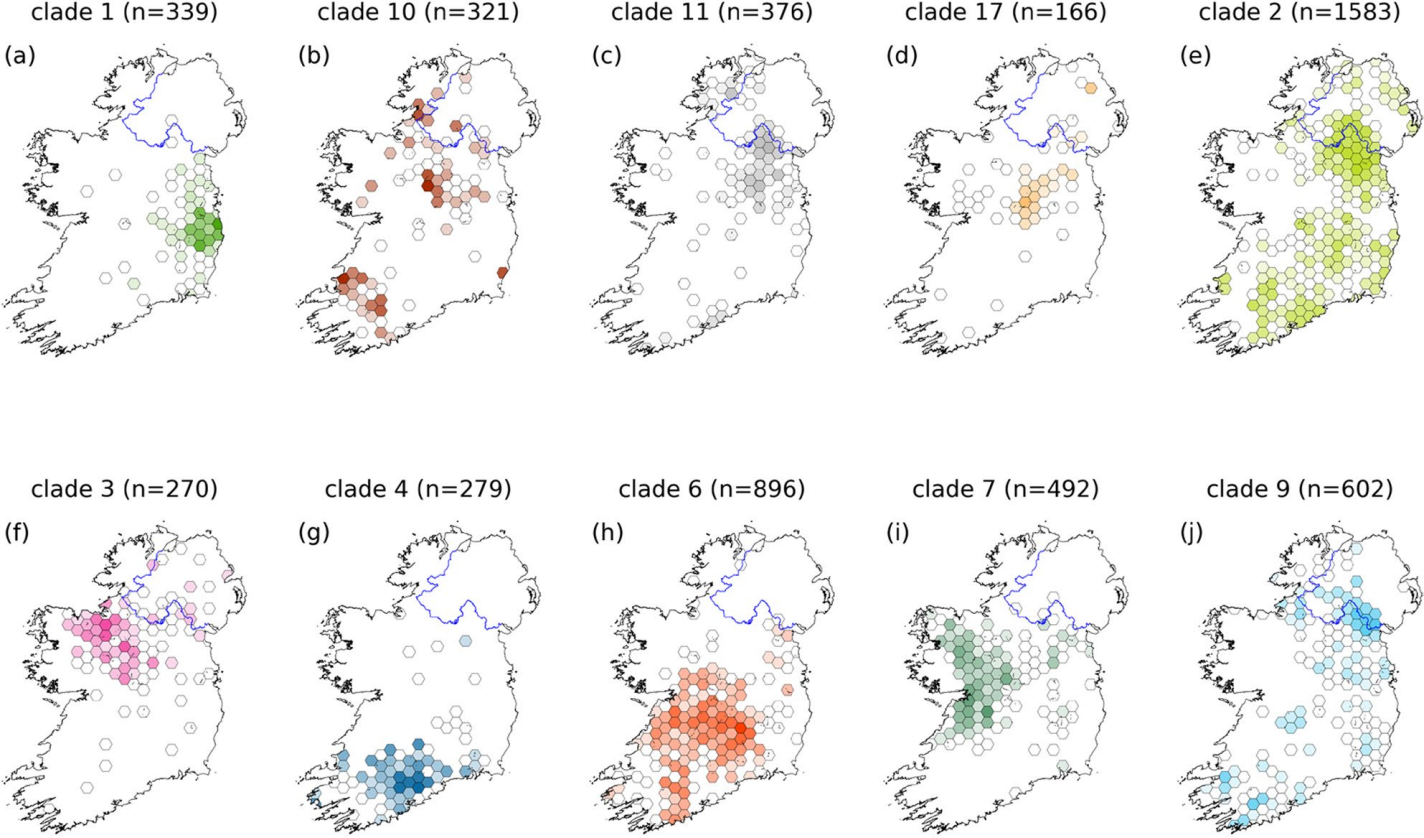
Hex bin plots showing the coverage of the 10 largest major clades (labelled (**a**)-(**j**)), indicating their regional localisation. 97% of the sampled isolates fall into these clades. Clade colours are the same as the tips in the phylogeny. The gradient reflects the number of samples in each bin

Major clades vary in their spatial extent and some show significant overlap. The spatial coverage of the 10 largest clades is shown in Fig. 4. The points belonging to each clade have been binned into hexagonal grids covering the island of Ireland. Small numbers of outliers have been removed for clarity where necessary. Bins are coloured by the number of counts in each bin, with the lowest value (1 sample) coloured white. Clades are given the same colours as in the phylogeny. Given the relatively uniform sampling across the island, this visualisation should represent the true range of these clades. These 10 clades constitute 97% of the sequenced isolates. Clade 1, centred around Co. Wicklow in Fig. 4(a) appears to have only limited spatial overlap with any other major clade. In contrast clade 2, Fig. 4(e) appears in much of the country though this clade can likely be further subdivided into two major clades when we formalise clade definitions. Note there is a discontinuity in some clades close to the border area between RoI and NI (i.e. clade 9 and 11). This is likely because we have high density of samples in NI at the border region around counties Fermanagh, Tyrone and Armagh.

### Within-species genetic diversity

**Fig. 5:**
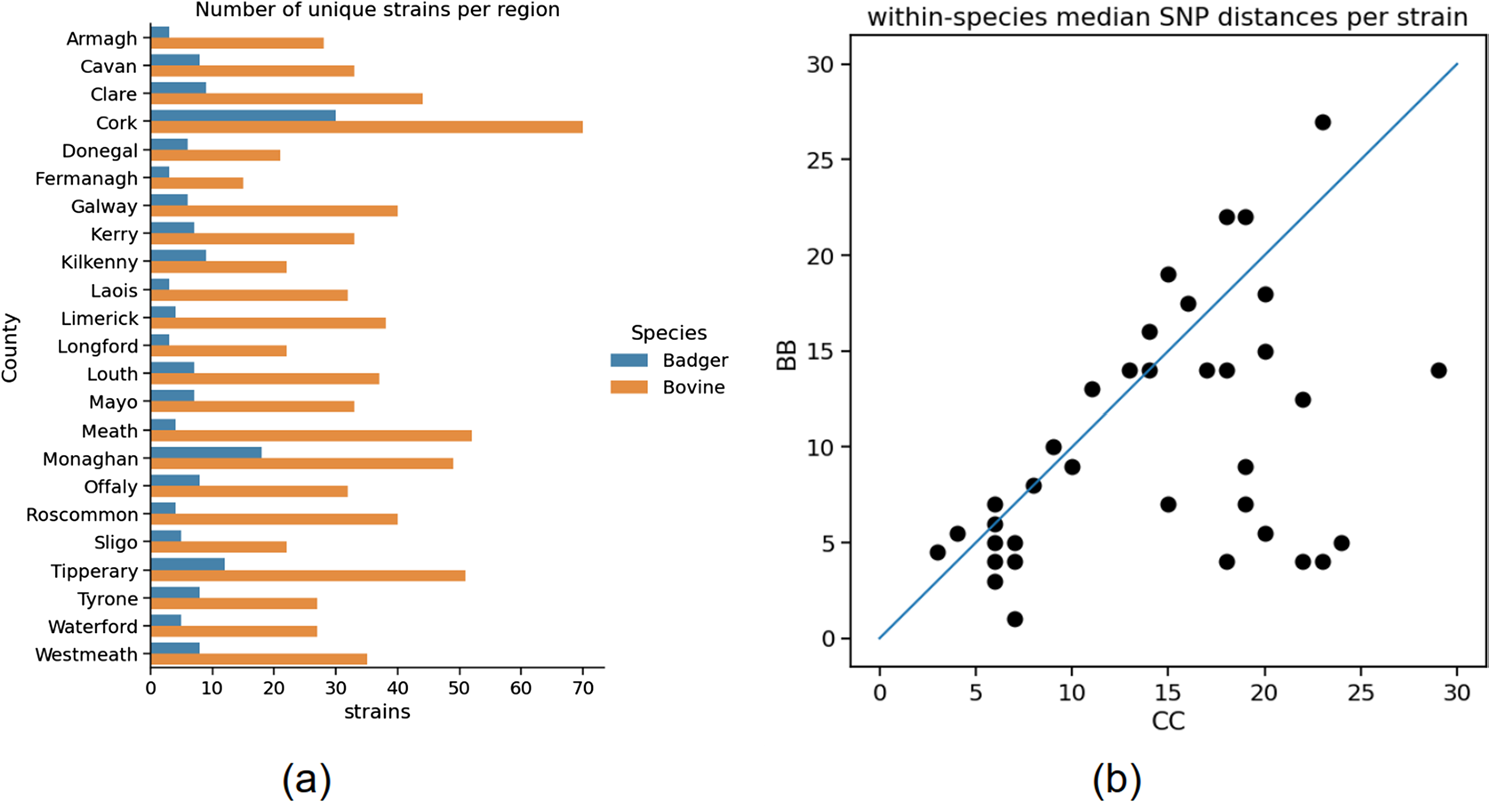
**a** The number of unique strains per county for cattle and badger. Unique strains are used as a proxy for strain richness, reflecting overall genetic diversity. **b** Correlation between the median pairwise SNP distance within cattle (CC) and badger (BB), per strain. Each point represents a strain (those with enough badger samples)

We first examined within-species diversity from a geographic standpoint. There is less genetic diversity in *M. bovis* isolates from badgers in any given area due to the lower spatial spread, as shown in Fig. 5(a) where the number of unique strains is used as a measure of diversity in each species. The plot shows unique strain counts on a per county basis, but this trend applies to any arbitrary area. Note that it is likely that the badger diversity is being systematically under estimated due to sparser sampling. There is a linear correlation between the number of samples and number of strains in an area (shown in Figure S4), which holds for both species. Whether this relationship breaks down or not at higher levels of badger sampling might be shown with more sampling.

SNP distances within species were also examined on a per strain basis. This avoids pairwise comparisons across major clades that would obscure the finer-scale patterns within epidemiologically relevant groups. Only strains that had at least five badger samples were considered. There is considerable correlation, as shown in Fig. 5(b), between the within-species genetic distance distributions in the same strain as would be expected. In some strains the badger-badger (BB) SNP distances are significantly lower than the corresponding cattle-cattle (CC) distances. These are the points below the diagonal. Again, this is possibly due in part to lower density or sporadic badger sampling.

### Spatial patterns at the strain level

**Fig. 6:**
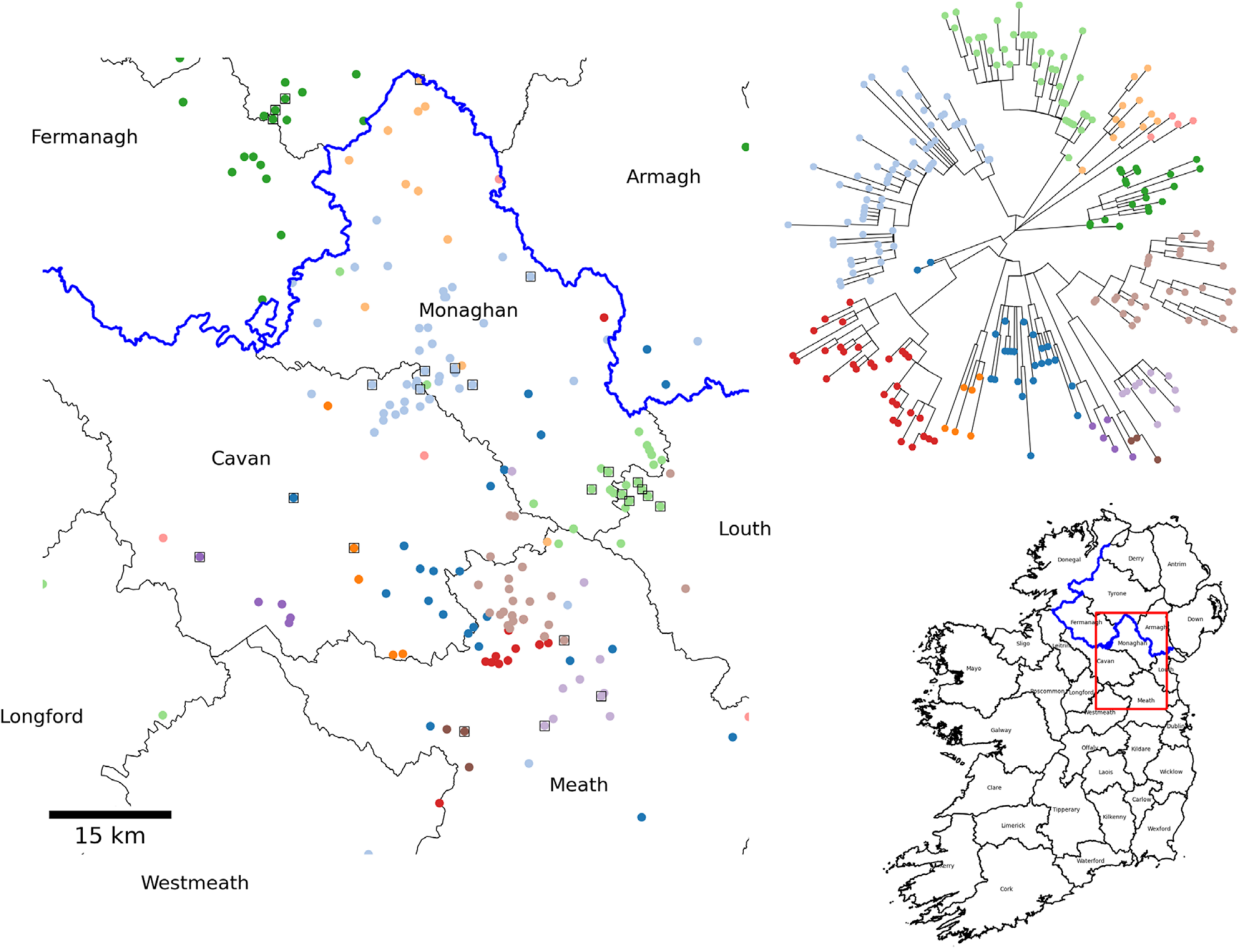
Spatial distributions of a well sampled strain centred around Co. Monaghan. The points are herd locations of the isolates coloured by cluster (threshold = 12 SNPs) which indicates that closely related groups within the strain are spatially clustered. Badgers are outlined with a square. The region is outlined by the red box in the map of Ireland, shown on the lower right. The upper right tree demonstrates the genetic relationship between the clusters

Our analysis reveals several recurring spatial patterns among *M. bovis s*train defining clades across Ireland, perhaps reflecting different transmission dynamics. Figure 6 illustrates a representative example of strain-level spatial structure for a strain distributed widely in the north-east of the country. Samples from the strain were further clustered using a 12-SNP distance threshold with each point being a herd or sett locations, coloured according to cluster. Localized clustering is evident, where distinct genetic sub-clusters remain geographically restricted. This suggests ongoing local transmission following initial introduction, with limited onward spread. In this case, clusters are shared between cattle and badgers, supporting the role of local wildlife in maintenance.

In contrast, other strains show diffuse dispersal across broader regions with extensive sub-cluster mixing. These patterns may reflect historical transmission involving frequent animal movement or shared grazing areas, leading to overlapping genetic signals. We also observe single-origin expansion patterns, where a concentrated cluster appears to seed genetically related outliers at a distance. This is consistent with recent dissemination events, potentially linked to cattle trade or high-risk cattle movements. Some clades exhibit low internal diversity with distant genetic outliers, possibly indicating recent emergence or a point-source introduction followed by spread. The presence of genetically similar strains in both cattle and wildlife in such cases may also point to recent cross-species transmission.

### A subset of herds carry multiple strains

**Fig. 7:**
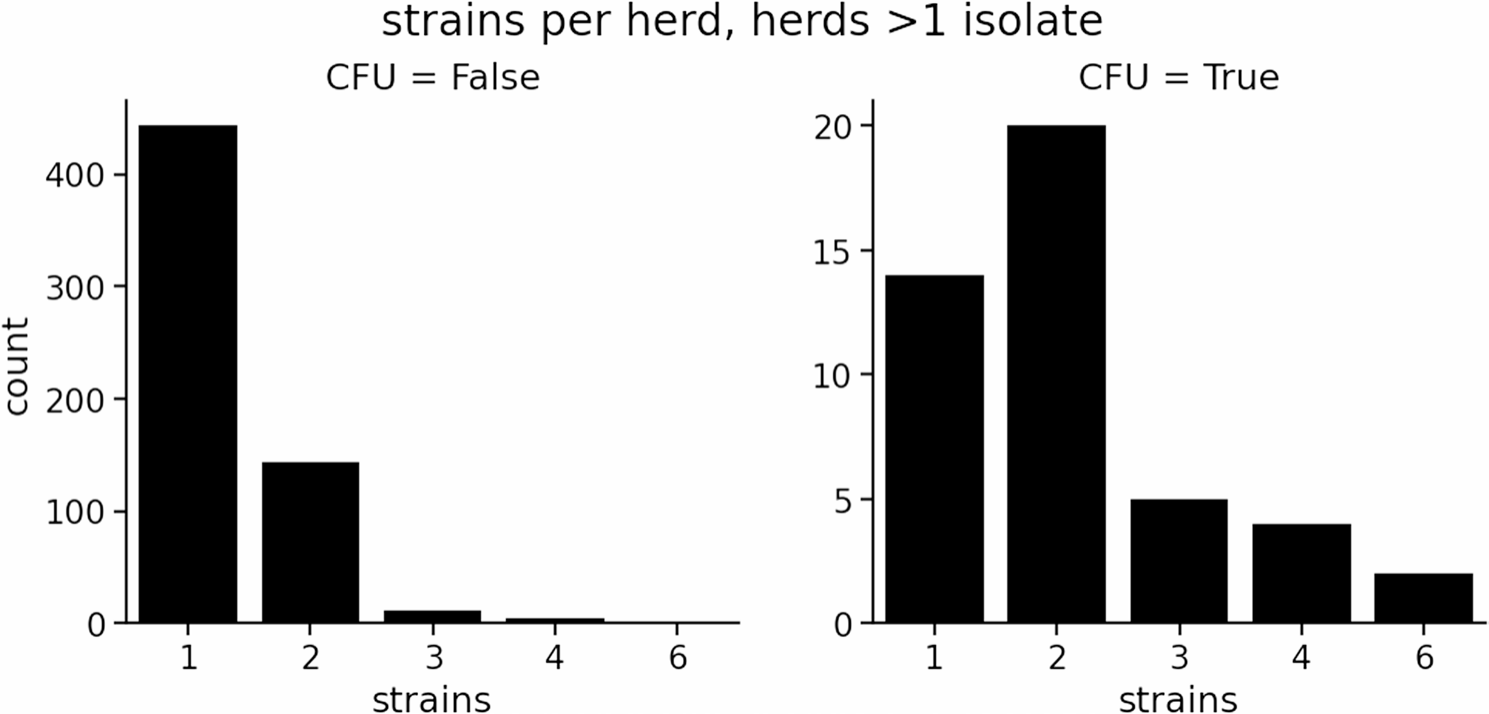
Numbers of sampled herds with two or more isolates (*n* = 648) that have multiple strains. The herds are split by CFU status (left and right plots) and show a marked difference in strain distribution

In our dataset 648 of the 2,962 RoI herds have had two or more isolates sequenced. Within herd diversity is generally low as seen in previous studies [41]. In 457 of these herds the isolates were the same strain with the median SNP distance ranging from 0 to 6, indicating recent shared transmission chains (e.g. strip plot in Figure S5(a)). The remaining 191 are those that contained at least 2 distinct strains. For these herds diversity is much larger depending on which particular strains are present. Sampling more than 2–3 isolates in a herd does not change these distributions (Figure S5(b)). Of the 191 multi-strain herds it is clear that those animals cannot have been infected in the same transmission event. All of these multiple strain herds are therefore of particular interest from a risk-based perspective. The distribution of the number of strains per herd is shown in Fig. 7. The plot is broken down by herd CFU status and demonstrates a markedly different distribution than other herd types.

### Controlled finishing units

**Fig. 8:**
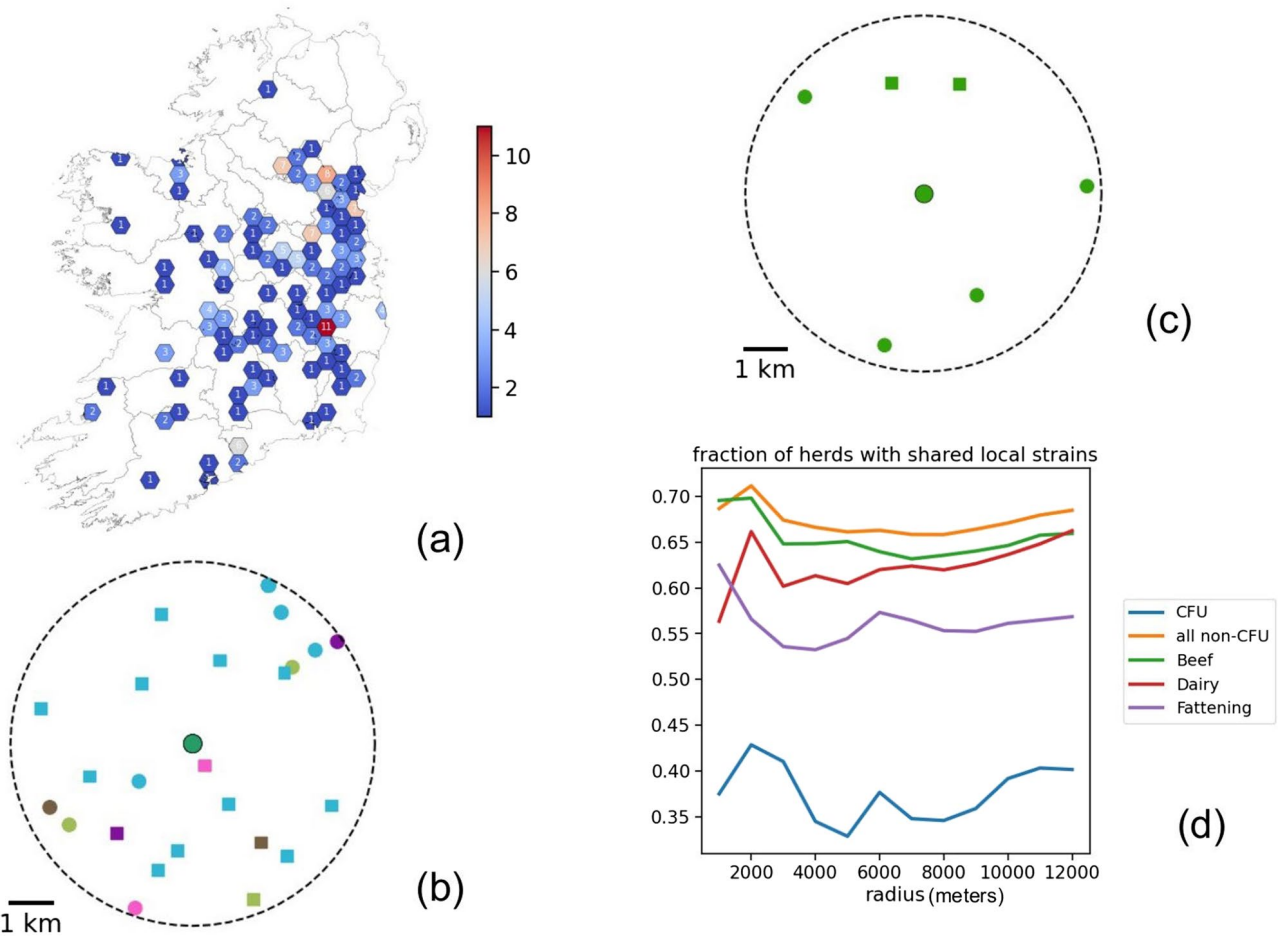
**a** Distribution of CFU herds in the dataset. **b** Example of a herd (centre point) with no shared local strains, threshold radius shown by circle. Points are coloured by strain and square points are badgers. **c** Opposite situation with the herd sharing the same strain as all it’s neighbours. (Points are the centroid location of the largest herd parcel or sett location). **d** Fraction of herds sharing local strains plotted against search radius for all CFU, non-CFU and other farms types

Since CFU status herds appear more likely to harbour multiple strains we wanted to further examine the tendency of these herds to share their strains with surrounding herds. CFUs were identified from our dataset by checking against a list of herds known to have had this status. In our dataset of 2,962 unique herds we found 148 that have (or have had) CFU status. The spatial distribution of these herds is given in Fig. 8(a). For all herds, neighbouring herds with samples were identified within a threshold radius of 5 km. Those with no neighbours were excluded. Strains in neighbours were then compared to the herd strains so that we could assess if any herd shares local strains (see methods section *Calculation of CFU association with local strains*). Figure 8(b) and (c) show the concept of local strain sharing. In (b) the herd (point at centre) shares no strain in common with its neighbours (points are coloured by strain and squares are badgers). Figure 8(c) is the opposite scenario where all neighbours are the same strain. From this analysis we could create a binary true/false ‘shared’ value for each herd. We then counted the number of herds with shared strains in neighbours between CFU and non-CFU herds. The results were put into a 2 × 2 contingency table shown in Table 1. Performing Fisher’s exact test on these values produces an odds ratio of 0.24 (95% CI 0.13–0.39), p-value: <0.00001. This indicates that CFU herds in our data are significantly less likely to share strains with neighbours. We also performed the same test using only multiple isolate samples to exclude the effect of only sampling one isolate and the result is similar with the odds ratio dropping to 0.13. Fitting a logistic regression model with ‘shared’ as the dependent variable also shows a significant negative association with CFU status (p-value = 0.001).

**Table 1:** Contingency table for number of herds in each category (CFU/non-CFU) that shared local strains at a distance threshold of 5 km

	CFU	Non-CFU
Shared	23 (32%)	1347 (66%)
Not shared	50 (68%)	705 (34%)

Finally, to test the effect of distance thresholds, we performed the analysis varying the threshold from 1 up to 12 km and then measured the proportion of shared/non-shared for each category. Herds were also split by herd class (beef, dairy and fattening). The result is shown in Fig. 8(d) with the fraction of herds with shared strains shown for each herd class. The fractions are consistent across thresholds and CFU herds are always lower. There also appears to be a distinct difference for fattening herds. Since we are only using a subset of herds, the CFU herds sampled (and with neighbours sampled) are still relatively small and this effect may not hold with more data. However, this is a strong indication of differences in local strain sharing for this class of herd.

## Discussion

This study is the first comprehensive, high-resolution analysis of the genetic diversity of *M. bovis* across the island of Ireland. We sampled a substantial portion of the overall strain diversity in the RoI and NI, with comprehensive spatial coverage of cattle isolates. Badger sampling tended to be more localised to areas with higher bTB burden and there is not yet uniform coverage across the country. As it currently stands there is relatively low temporal depth in the dataset, with about 700 isolates of the total 5875 isolates sampled prior to 2017 (the majority of these are from NI). Our results reveal a large overlap between RoI and NI isolates, with the latter being mostly a subset of the overall diversity across the island. Using representative strains from GB we see that most GB strains fit inside the island of Ireland phylogeny, with the exception of two clades that are not present in Ireland. SB0140 is the dominant spoligotype in Ireland, but its appearance in multiple parts of the phylogenetic tree reflects homoplasy and highlights the limited resolution of spoligotyping compared to SNP-based analyses.

Major clades were generally found to be regionally localised. This is consistent with observed patterns seen in GB [42] and recently reported in Wales [43]. Analysis of spatial patterns within strains revealed that in some cases, sub-clusters also exhibit strong spatial localization, supporting evidence of local transmission and recent spread, where closely related strains are confined to specific geographic regions. However, some strains display a high degree of genetic similarity despite being widely dispersed, indicating long-distance movement events or multiple introductions of closely related strains. When sampling density is high enough badgers are frequently found to share strains with cattle, which indicates transmission of infection between species. Further phylodynamic studies would be needed to ascertain the direction of transmission on a case-by-case basis. While these spatial structures offer insights into underlying epidemiological processes, we recognise that some patterns may be influenced by sampling density and distribution. Incompletely sampled regions, over-representation of specific herd types, or gaps in wildlife surveillance may affect how strain dispersion appears. Ongoing integration of movement data, land use, and wildlife monitoring will help clarify whether observed patterns reflect true transmission dynamics or artefacts of sampling.

Our analysis reveals notable patterns of within- and between-host species diversity. Isolates from cattle exhibited a higher overall diversity compared to those from badgers, as evidenced by the greater number of unique strains per geographical unit (i.e. county). This likely reflects both the broader geographic range and greater movement of cattle, which increases opportunities for exposure to diverse strains. In contrast, badger populations appear to host fewer, more geographically localized strains. When comparing host-specific genetic diversity within strains, we found a strong correlation between cattle and badger median pairwise SNP distances, suggesting that genetic diversity trends are generally conserved across hosts. However, in several strains, badger-badger distances were lower than cattle-cattle distances, indicating that transmission among badgers may be more localized, consistent with their territorial behaviour and limited dispersal. These findings support the view that badgers act as stable local reservoirs of *M. bovis*, while cattle serve as a more dynamic component of the transmission network, influenced by long-distance movements and herd-level epidemiological factors.

The presence of multi-strain herds, where multiple distinct strains are detected within a single herd, appears to be relatively common with 30% of herds having more than one strain present (*n* = 191 out of 648 sampled herds). This highlights the value of sequencing more than one isolate in a herd when conducting epidemiological investigations. These herds must have acquired different strains through multiple introductions rather than a single transmission event, suggesting a potentially higher risk profile for those herds. One important factor may be undetected residual infection that has persisted from a previous breakdown, with a new strain introduced later. Understanding the drivers of multi-strain persistence through genomic surveillance may help refine approached like targeted testing strategies in the future.

CFU herds offer a unique opportunity to study the comparative genomics of different herd classes. Our results show that CFUs are more likely than all other herd types to contain multiple strains (within the time limits of our sampling). By comparing strains isolated from cattle within CFUs to those found in surrounding herds, we can also determine whether CFUs predominantly house strains endemic to the local area or strains from outside their typical home ranges. Our findings suggest that CFUs tend to contain strains that are less commonly found in surrounding herds, reinforcing the hypothesis that they receive non-local strains through inward cattle movements rather than reflecting local epidemiology. This analysis can be improved with greater numbers of sequenced samples from CFU herds, where isolated cultures from infected herds become available. Such approaches are made challenging by the many various confounding factors that contribute to transmission, not the least of which is sample availability. We have also made certain simplifications to facilitate this preliminary analysis. For example, centroids of all land fragments associated with the herd were used, ignoring individual fragments. Furthermore, the majority (65%) of our cattle isolates are from animals with no moves prior to slaughter. CFUs in our dataset tend to have substantially more inward animal movements compared to other herd types. In other words, the apparent effect of CFU status may partly reflect the influence of higher movement volume, which is itself associated with lower likelihood of local strain sharing (possibly due to greater strain diversity or dilution of local strains). However, this approach is promising and we aim to refine the methodology going forward.

While mostly descriptive in nature, our work provides a foundation for utilising WGS and integrating it with location across the island of Ireland to yield insights on the spatial pattern of strains and transmission dynamics. The integration of ‘home range’ analyses into *M. bovis* epidemiology offers several promising directions for future research. Our definition of home ranges in this study was mainly used for CFU analysis. This differs from the definition applied in GB [44] where it is used in for routine surveillance and would have to be refined in a similar way for the Irish context. By refining these analyses, such as defining strain home ranges using temporal patterns of outbreaks and excluding high-risk movement herds, we can establish a clearer baseline for endemic strain circulation. Future work could leverage home ranges to identify the presence of a strain outside its defined home range and serve as an indicator of long-distance transmission trends. Future research could also systematically explore temporal shifts in strain home ranges, particularly by increasing historical sampling depth to detect meaningful evolutionary or epidemiological trends. Systematic WGS of *M. bovis* only began in 2019 (with lower amounts from 2017) and there is still relatively low temporal depth in the dataset, such that phylodynamic studies are only now starting to become feasible. This situation should improve in the near future. It may also be possible for priority to be given to sequencing of retrospective isolates in the DAFM laboratories archive that would broaden the temporal depth of the current genomic data.

This work highlights the utility of combining genomics with the epidemiological context, namely the spatial, temporal and contact relationships between the sampled hosts. Work is ongoing to integrate land parcel, testing and movement data in more sophisticated ways to expand the use of WGS. To this end we have developed an experimental interactive web application that will aid in the exploration of this data, called tracebTB [45]. A platform such as this would also have major utility in sharing of results with stakeholders and programme managers at local and national level.

Finally it is planned to formalise the provisional strain nomenclature used here along the lines of that implemented by APHA for England and Wales [44].

## Conclusion

This study provides a high-resolution genomic descriptive analysis of *M. bovis* across the island of Ireland, integrating WGS with spatial mapping. Our findings reveal significant regional variation in strain distribution, with some clades remaining highly localized while others exhibit broader dissemination, likely driven by cattle movements. The genetic similarities between cattle and badger isolates reaffirm the role of badgers as a key wildlife reservoir. These findings emphasize the need for a more refined, risk-based approach to bTB control in Ireland. Integrating WGS into routine surveillance could enhance early detection, improve outbreak tracing, and inform targeted interventions, particularly in high-prevalence regions. By combining genomic insights with movement data and host ecology, bTB control measures can be better tailored to disrupt transmission pathways and reduce disease persistence. As the RoI and NI continue to invest in bTB eradication, the application of genomic epidemiology will be crucial in refining strategies, minimizing economic losses, and ultimately achieving disease elimination.

## Supplementary Information

Supplementary Material 1.

Supplementary Material 2.

Supplementary Material 3.

## Data Availability

Raw sequence data for 72 representative sequences is available on the Sequence Read Archive at bioproject PRJNA1279664. Relevant code used in this analysis is available in a github repository at https://github.com/dmnfarrell/gordon-group/tree/master/ireland_btb_wgs.

## Abbreviations

WGS: Whole genome sequencing
SNP: Single nucleotide polymorphism
M. bovis: Mycobacterium bovis
CFU: Controlled finishing unit
