# Supplementary material for: The genomic diversity and spatial patterns of Mycobacterium bovis in Ireland revealed by whole genome sequencing
Source: Ir Vet J. 2025 Dec 3;79:6. doi: 10.1186/s13620-025-00324-0 (PMC12781239; doi:10.1186/s13620-025-00324-0)
Supplement: Supplementary file 1 — Supplementary Material 1. [file 13620_2025_324_MOESM1_ESM.pdf]

The diversity and spatial patterns of *M.bovis* in Ireland revealed by whole genome sequencing.

Supplementary Material

James O'Shaughnessy[1], Nicola Harvey[2], Brian Byrne[1], Máire McElroy[1], Montserrat Gutierrez[1], Declan Murphy[1], Kevin Kenny[1], Henrietta Cameron[1], Deirdre Prendergast[1], Rebecca Cupial[1], Margaret Goggin[1], Lionel Kenneth Dygico[1], Jordy Smith[3], Jamie A Tratalos[2], Ryan Devaney[4], Purnika Ranasinghe[4], Tara Ardis[4], Adrian Allen[4], Guy McGrath[2], Stephen V. Gordon[3,5,6], Damien Farrell[3 ¶]

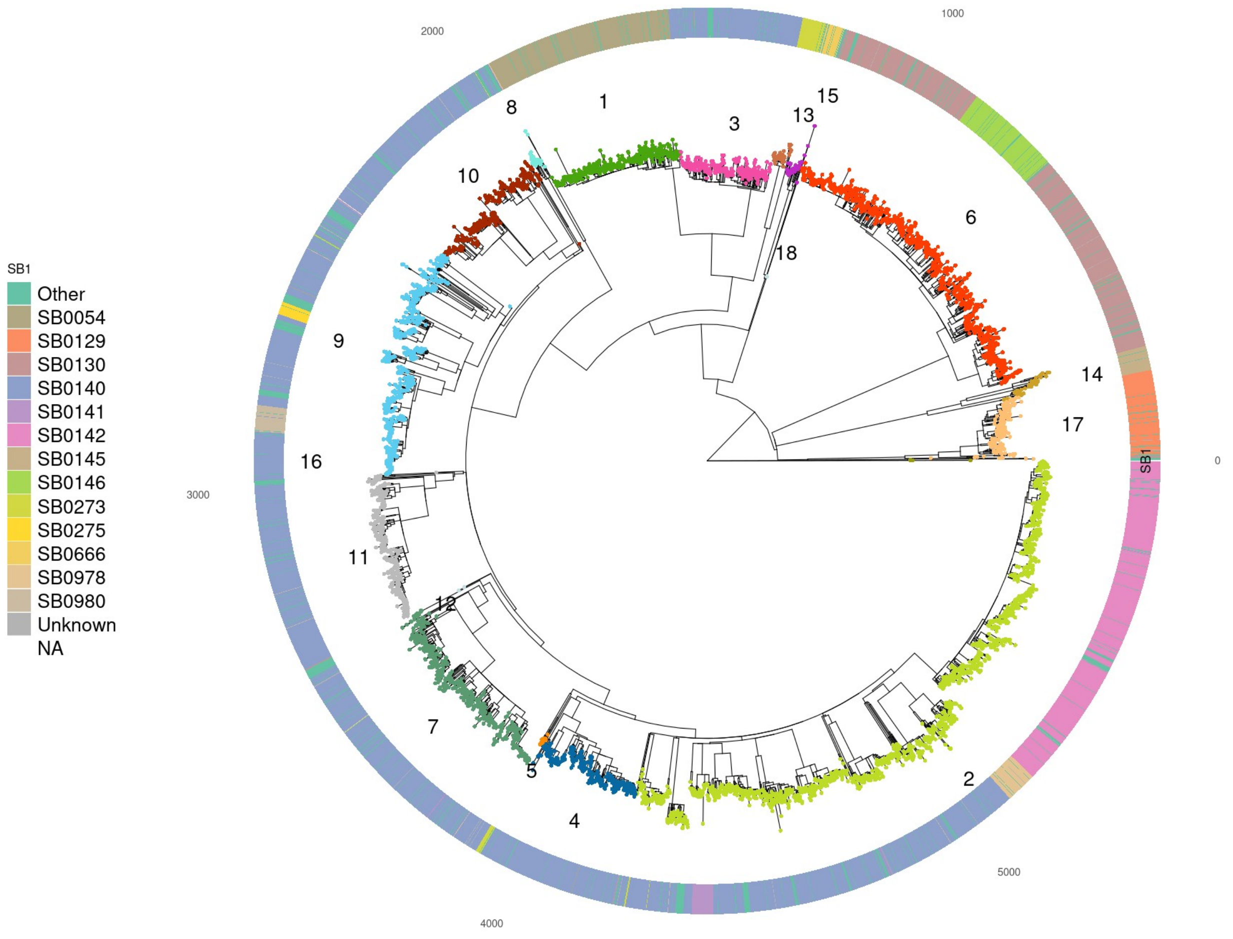

Figure S1: Phylogeny of all samples indicating the main lineages of M. bovis found in the study. Tips are coloured by their lineage which are also numbered. The outer ring is a heatmap indicating spoligotype.

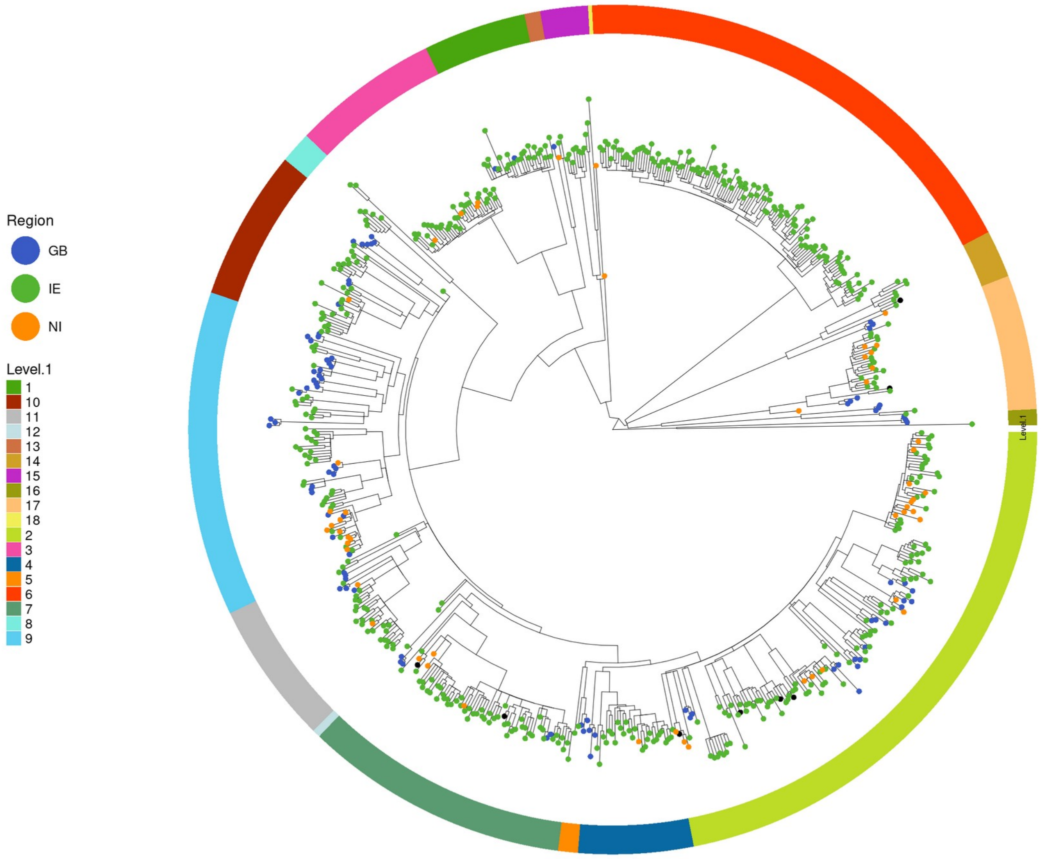

Figure S2: Phylogeny of representative samples with all GB samples included to show where they lie in the tree.

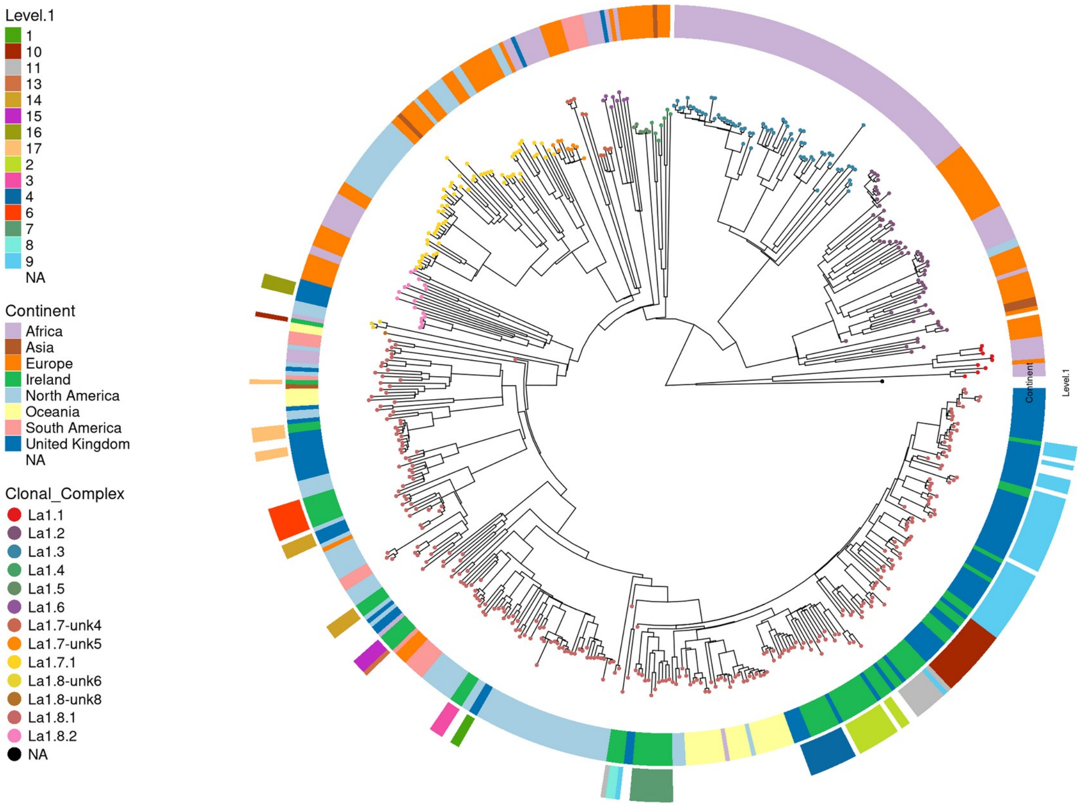

Figure S3: M. bovis global phylogeny showing the position of selected Ireland and UK samples in the La1.8.1 (Eu1) lineage. Tips are coloured by the clonal complex identified by Zwyer et al. Continent (with Ireland and UK separate) is shown in the inner ring and the Irish clades identified in this study are shown in the outer ring, coloured by major clade as in the main paper.

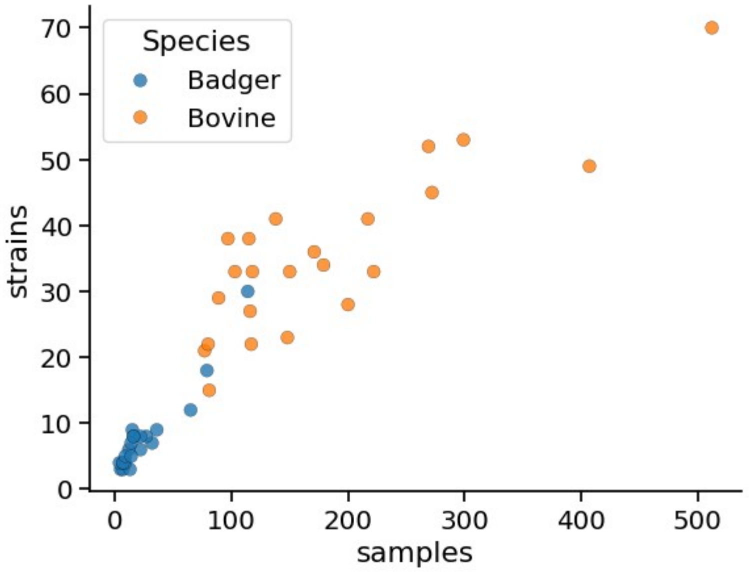

Figure S4: There is a linear relationship between the number of samples and strains in a given area. This holds for both species including the points where badger sampling is higher.

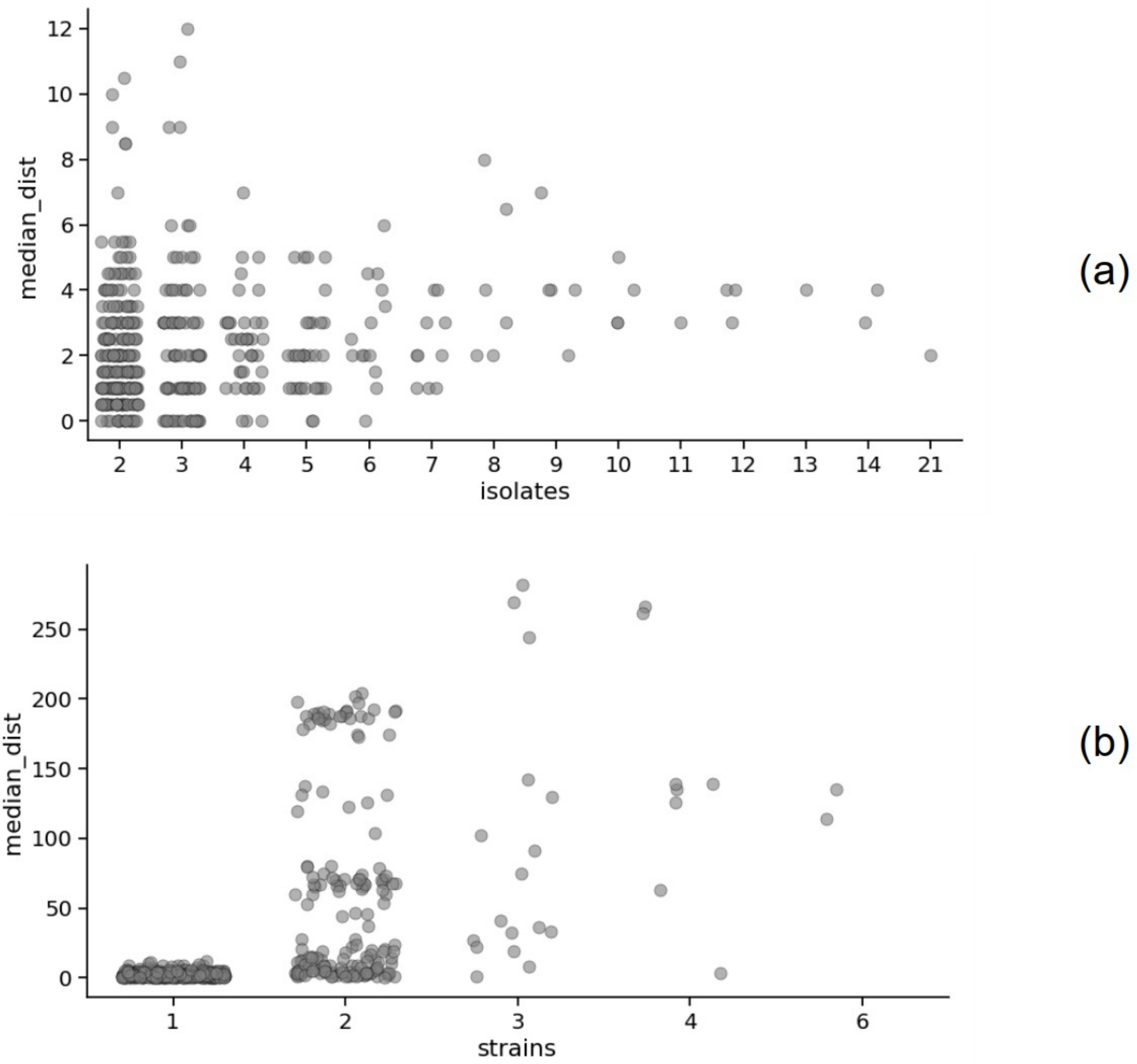

Figure S5: Strip plots showing distributions of median SNP distances in herds with >1 isolate. There were 457 such herds in all. (a) Those herds with 1 strain plotted against no. of isolates sequenced. (b) Median SNP distance vs number of strains per herd showing a large variation in distances for those with more than one strain present.
